# The Effect of Age and Use of Enamel Matrix Derivative on Implant Loss

**DOI:** 10.3390/dj14010063

**Published:** 2026-01-19

**Authors:** Stephen K. Harrel, Thomas G. Wilson, Martha E. Nunn, Charles M. Cobb

**Affiliations:** 1 Department of Periodontics, Texas A&M University College of Dentistry, 3302 Gaston Ave, Dallas, TX 75246, USA; 2 Private Practice, Dallas, TX 75231, USA; tom@northdallasdh.com; 3 Private Practice Dentistry and Biostatistics, Omaha, NE 68127, USA; menunn@gmail.com; 4 Department of Periodontics, School of Dentistry, University of Missouri-Kansas City, Kansas City, MO 64108, USA; cobbc@umkc.edu

**Keywords:** implant loss, peri-implantitis, enamel matrix derivative, age, biologic agent

## Abstract

**Background:** Previous reports suggest that patient age at the time of implant placement is not a factor in implant survival. However, analysis of data compiled for a previously published study on the effect of enamel matrix derivative (EMD), a frequently used biomaterial to aid bone regeneration, on peri-implantitis indicated that age and use of EMD may be a factor in implant survival. The current study further evaluated the existing database to determine the effect of age and EMD use on long term survival of implants. **Methods:** An existing database from a private periodontal specialty practice was evaluated for the effect of age at the time of implant placement on implant survival. In addition, all available clinical factors were evaluated, including the use of EMD at any point during site preparation or implant placement to determine any effect on implant survival. **Results:** Patient age at the time of implant placement had a negligible effect on implant survival for younger individuals. However, starting at 58 years of age, an increase in relative risk for implant loss was noted. When the patient age was divided into groups, it was determined that patients ≥ 58 and ≤68 years had a statistically significantly increased relative risk of implant loss (2.75), which was sharply reduced if EMD had been used (1.24). This trend was also noted to a lesser extent in patients older than 68 years. **Conclusions:** The risk of implant loss was elevated when implants were placed in older patients. This risk was reduced if EMD had been used at any point during implant site preparation or placement.

## 1. Introduction

Despite the relatively high success rates of dental implants, peri-implantitis remains the most common complication, which often leads to implant failure [1,2,3,4]. Depending on the definition of peri-implantitis, studies report that between 15.2% [5] and 19.5% [6] of implant patients develop peri-implantitis, or at the implant level, between 8.7% [5] and 12.5% [6] of implants will exhibit peri-implantitis. Derks et al. [7] reported a 45% prevalence rate of peri-implantitis in patients at 9 years post-insertion. In this same group of 588 randomly selected patients, the prevalence of moderate to severe peri-implantitis was reported at 14.5%.

While there may be debate on the percentage of patients that will suffer from peri-implantitis, the total number of implant failures will rise with the placement of more fixtures and with increased time of function. There is no totally predictable treatment for implants that are failing due to peri-implantitis [8,9,10]. While surgical treatment for peri-implantitis has shown to be more effective than various non-surgical approaches [9], failures still occur [11,12,13,14]. Based on the potential for implant failure, any adjunctive therapy incorporated during the implant placement procedure that may suppress the development of peri-implantitis and implant loss will have long-term patient benefit.

The possibility that enamel matrix derivative (EMD), a frequently used biomaterial for bone regeneration in use for over 30 years, might have a positive effect on implant retention came to light in a recently published case series study that evaluated the effect of utilizing EMD during the treatment of peri-implantitis [15]. The study reported a high retention rate of implants and a significant improvement (*p* = 0.001) in pocket probing depth when EMD was used as part of treatment for peri-implantitis. The data collected for the study also indicated a trend for the potential reduction in implant loss in older individuals when EMD was used at any point during initial implant placement. This trend was not the focus of the initial study and, consequently, was not reported. As a follow-up to our previous study, the trend for reduced implant loss when EMD is utilized is further evaluated in the current study.

Traditional factors known to have a negative effect on implant retention and the occurrence of peri-implantitis have included smoking, presence or history of periodontitis, systemic factors such as diabetes, iatrogenic factors, and potentially other factors [16,17,18,19]. Notably, there are few papers dealing with the effect of age on implant retention and failure. While there are indications that age is not a relative risk for implant loss [20,21], it should be noted that the ability to heal after injury diminishes with age [22,23]. The reasons for this lessened healing ability and/or slower rate of healing appear to be multifactorial. The reduction in the number of available stem cells as individuals age [24,25], as well as other factors [26,27], could conceivably impact both initial healing and the long-term retention of implants [22,23]. Age is also associated with an increase in the amount and severity of periodontitis, which has been associated with exacerbating peri-implantitis [28,29].

Given the lack of a predictable treatment for peri-implantitis, any adjunctive therapy used during implant site preparation and/or placement that may improve long term outcomes and reduce implant loss should be explored. Unpublished data from our previous study [15] demonstrated a positive trend in implant retention in older patient groups in which EMD was used in the treatment of peri-implantitis. Thus, the purpose of this study is to determine if there is a relationship between the age of the patient at the time of implant placement and implant loss. A secondary purpose is to determine if the use of EMD during any phase of implant placement has a relationship to implant loss in different age groups of patients.

## 2. Materials and Methods

This is a retrospective study taken from a database of dental implants generated in a private practice of periodontics. All procedures were performed by experienced periodontists. The study was performed in accordance with the Declaration of Helsinki and Good Clinical Practices. While not a clinical trial, it was registered with ClinicalTrials.gov to comply with university recommendations (identifier NCT05419102). The protocol for preparing the database and reporting the clinical results was approved by the Center for IRB Intelligence (Pro00059785, approval date: 6 January 2022) and complied with HIPAA requirements. This is the second study using this database [15]. All patients gave written permission for their clinical information to be used for scientific studies.

The implant database used in this study was prepared for a previously published study that evaluated the effect of using EMD in the surgical treatment of peri-implantitis [15]. In preparation for this previously published study, all implants in which EMD was used during placement or in the treatment of peri-implantitis were evaluated. However, the published paper reported only the results of the use of EMD in the treatment of peri-implantitis.

A power analysis, detailed in the statistical analysis section, indicated that 150 patients with a 20% failure rate (30 patients with implants lost) would be necessary to determine if there was a relationship between EMD usage and age as a factor in successful retention of implants. Due to the high success rate of implants, developing an adequately powered study to statistically evaluate the cause of implant loss in a specific group, such as implants that had EMD used as part of their placement, is difficult. The low number of failures within any subgroup made it impossible to statistically evaluate the influence of the age of the patient on the relative risk of non-retention of implants using traditional methods. Because of this, the statistical method of oversampling was utilized [30]. Oversampling is increasingly used in certain groups or results, which allows for better estimation of the effect of specific factors in a group. This is followed by the use of sampling weights in analyses to avoid unintended biases, such as selection bias, associated with oversampling.

Because the power analysis indicated there needed to be a minimum of 20% implant loss within 150 patients, it was determined to use implant loss as the criterion for inclusion in the subgroup database. In order to have an adequate number (30) of patients with lost implants, the first 30 sequential patients who experienced implant loss were included in the database. Using a similar pattern of including sequential patients, the first 120 patients who did not experience implant loss were included in order to yield a database of 150 patients, within which 20% of the patients experienced implant loss (81 of 386 implants lost). Thus, the final database contained 386 implants with 305 successful implants (79.0%) and 81 failed implants (21.0%).

For the current study, the database of 386 implants was searched for all patients who had EMD used during any step of their implant placement (73 implants, 26%). This included the use of EMD during extraction site preservation, guided bone regeneration to prepare the site, or during the placement of the implant. Thus, the use of EMD was for the most part used in patients with inadequate native bone volume, those with a bony dehiscence, or those receiving immediate implantation. This dataset was evaluated for non-retention of implants based on multiple factors, including pocket depth, immediate placement, retention of prosthesis (cemented or screw retained), and other clinical factors. The data set was also evaluated for any relationship of patient age at the time of implant placement and the retention (non-loss) of the implant, any relationship to the use of EMD during implant preparation or placement to implant retention, and any relationship to EMD impacting implant retention in relation to patient age at the time of implant placement.

### Statistical Methods

Prior to the initiation of this study, sample size calculations were conducted based on both a simple survival analysis and a clustered survival analysis. In order to obtain 85% power to detect a significant hazard ratio of 1.5 at a 0.05 level of significance, a total sample size of 150 subjects with a minimum of 20% failure was required. Because the implant loss rate for most periodontists is generally less than 5%, it was necessary to oversample failures without regard for the primary predictor variables: EMD, bone graft, and immediate implant status. The total sample size was 150 subjects with 386 implants placed.

All statistical analyses were conducted using SAS 9.4 (SAS Institute, Cary, NC, USA), with adjustment for clustered data where necessary. Subject-level descriptive statistics were tabulated, and implant-level descriptive statistics were also calculated. The method of generalized estimating equations (GEE) was utilized to compare mean probing depth, deepest probing depth, and bleeding on probing (BOP) for subject-level and implant-level variables. Linear GEE modeling was used for mean probing depth and deepest probing depth. Logistic GEE modeling was used for bleeding on probing.

Frailty survival regression models were utilized to estimate the hazard ratio for implant failure for various subject-level and implant-level variables. Age was tested as both a confounder and an effect modifier in the modeling.

## 3. Results

Summary statistics were calculated for all available subject-level variables and are shown in Table 1.

Summary statistics were calculated for implant level variables and are shown in Table 2, GEE regression modeling was utilized to compare mean probing depth, deepest probing depth, and bleeding on probing by the various subject-level and implant-level variables, with the results shown in Table 3.

Frailty survival analysis was conducted on all available variables individually while adjusting for age in categories with relative risks, 95% confidence intervals for relative risks (RR), and associated *p*-values shown in Table 4. Age (groups: 16 to 58 years, >58 to 68 years, >68 to 88 years), diabetes mellitus status (yes vs. no), oral hygiene status (unsatisfactory vs. satisfactory), periodontitis status (periodontitis versus no periodontitis), and presence of peri-mucositis/peri-implantitis (peri-mucositis/peri-implantitis vs. no peri-mucositis/peri-implantitis) are all statistically significant at α < 0.05 level of significance. With age, the risk of implant failure was evaluated on a yearly basis. An increase in implant failure was noted at greater than 58 years of age, with those between >58 years of age and 68 years of age having 2.3 times the risk of implant failure compared to those ≤58 years of age. Patients older than 68 years of age were noted to have a slightly lower risk of implant failure than those in the >58 to 68-year age range. This group had 1.9 times the risk of implant failure compared to those ≤58 years of age. Smokers have 2.1 times the risk of implant failure compared to non-smokers, although this risk failed to achieve statistical significance (*p* = 0.069, RR = 2.12, 95% CI: 0.95 to 4.73). Subjects with diabetes mellitus have 3.1 times the risk of implant failure compared to subjects without diabetes mellitus (*p* = 0.019, RR = 3.10, 95% CI: 1.01 to 9.46). Subjects with unsatisfactory oral hygiene have almost 3.2 times the risk of implant failure compared to subjects with satisfactory oral hygiene (*p* = 0.031, RR = 3.15, 95% CI: 1.11 to 8.90). Subjects with periodontitis have almost 2.5 times the risk of implant failure compared to subjects without periodontitis (*p* = 0.014, RR = 2.46, 95% CI: 1.06 to 5.64). Implants with the presence of peri-mucositis or peri-implantitis have almost 6 times the risk of implant failure compared to implants without peri-mucositis or peri-implantitis (*p* < 0.001, RR = 5.94, 95% CI: 3.12 to 11.3).

Frailty survival models were also fit for all available variables individually while adjusting for age as a continuous variable with relative risks, 95% confidence intervals for relative risks, and associated *p*-values shown in Table 5. Similar trends are seen in Table 5 as are seen in Table 4. The primary difference is that age as a continuous variable fails to achieve statistical significance (*p* = 0.117, RR = 1.02, 95% CI: 1.00 to 1.05).

Effect modification of EMD by age, deepest probing depth (in categories) by age, and mean probing depth (in categories) by age were tested, but all models failed to achieve statistical significance. However, age (in categories) when analyzed by EMD status with the model for implants not treated with EMD showing a statistically significant association of implant loss to age (in categories) (*p* = 0.047) while the model for implants treated with EMD failed to show a statistically significant association between implant loss and age (in categories) (*p* = 0.557) as seen in Table 6. Age (in groups) was analyzed by deepest probing depth (in categories), and age was analyzed by mean probing depth (in groups), but neither set of models showed any statistical significance, as seen in Table 6.

## 4. Discussion

As shown in Table 1, Table 2 and Table 3, the expanded database used in this study yielded similar results to information we previously found when only the EMD peri-implantitis subgroup was evaluated [15]. This data reflects that the traditional risk factors of smoking, diabetes, and poor oral hygiene were associated with increased pockets and loss of attachment. The summary clinical data indicate there is no difference in clinical measurements, such as pocket depth and attachment levels, between when EMD is used and when not used. The clinical findings were similar when comparing non-oversampled vs. oversampled databases. This observation indicates that, despite inherent selection bias, using an oversampling technique to expand the ability to evaluate a specific therapy is a viable approach to the frequently encountered problem of an inadequate number of failed implants within a subgroup.

When the oversampled database was evaluated for the relative risk of implant loss by age, there was an overall increased relative risk for implant loss with increased age. In the younger age group (≤58 years), the increase in the risk of implant loss with age held true regardless of use or nonuse of EMD. In cases where EMD was not used in patients >58 years of age, the trend towards an increased relative risk for implant loss remained. However, there was a sharp decrease in the relative risk of implant loss with the use of EMD vs. the nonuse of EMD.

This indicates that the use of EMD during implant preparation or placement appears to diminish the risk of implant loss in patients older than 58 years.

The reason for the sharp reduction in the relative risk of implant loss when EMD is used in an older group is unknown. The effect of age and healing has been studied extensively in the dental and medical literature [20,21,22,23,24,25,26,27,28,29]. Various changes in healing with age, such as cell senescence and a decrease in inflammatory signaling, have been proposed, but the exact mechanism(s) are unknown. Postulating the role of EMD in reducing implant loss must remain speculative. It is possible that EMD has a direct positive influence on the initial healing of the implant site, or it may be that EMD stimulates the production of healing factors which naturally diminish with age [31,32,33]. The fact that EMD use did not reduce the relative risk of implant loss in younger patients vs. the relative risk reduction in older individuals suggests that the use of EMD may replace or stimulate factors that are lost or diminished with aging.

There are several potential weaknesses in the present study. The first is the use of a retrospective database. While a prospective blinded study is the gold standard for determining clinical results, the American Academy of Periodontology and the European Federation of Periodontology have indicated that a prospective study of implant loss would be unethical [34].

Another potential weakness is the use of relative risk as an endpoint for analysis. Relative risk gives an indication of the possibility of an occurrence, in this case, implant loss, but it is an estimate of the implant being lost as opposed to a direct measure. While our findings that EMD will reduce implant loss are statistically significant, there is the question of whether EMD use in older patients is clinically significant [35,36]. As clinicians, clinical significance is the empirical endpoint that we are striving for in our patients’ treatment. Clinical relevance will require a larger, more controlled study.

A final potential weakness is the use of oversampling to adequately power a database so that statistical analysis can be performed. This technique is increasingly used in medicine to address data disparities similar to what is seen in implant loss [37]. Oversampling has an inherent selection bias in the fact that it utilizes subjects that are not uniform. In the current database, EMD was used in situations where there was usually less than ideal bone volume, thin or damaged cortical plates, or the placement of an immediate implant. These uses of EMD represent a further selection bias, as EMD was not used in ideal circumstances but in more challenging cases that were more likely to fail.

## 5. Conclusions

Within the limitations of this study, there is a trend towards increased implant loss with increased age of the patient at the time of implant placement. The use of EMD during implant site preparation and/or implant placement did not seem to impact clinical parameters such as pocket depth or attachment levels. The use of EMD also did not appear to impact the relative risk of implant loss, except in older individuals. In patients >58 years of age, the use of EMD sharply reduced the relative risk of implant loss. These results are the first report of the potential of EMD to decrease implant loss in older individuals. These results need to be further evaluated with a larger sample size from a more controlled database.

## Figures and Tables

**Table 1:** Summary statistics for all available patient-level variables.

Variable	
**Age**	
** n**	150
** Mean ± SD**	62.7 ± 11.7
** Median**	63.5
** Minimum**	16
** Maximum**	88
**Gender**	
** n**	150
** Male**	43.3% (65/150)
** Female**	56.7% (85/150)
**Diabetes Mellitus?**	
** n**	150
** No**	94.0% (141/150)
** Yes**	6.0% (9/150)
**Smoking Status**	
** n**	139
** Nonsmoker**	84.9% (118/139)
** Smoker**	15.1% (21/139)
**Hygiene Status**	
** n**	147
** Satisfactory**	94.6% (139/147)
** Unsatisfactory**	5.4% (8/147)
**Periodontal Disease on Natural Teeth?**	
** n**	107
** No**	79.4% (85/107)
** Yes**	20.6% (22/107)
**Nightguard?**	
** n**	150
** No**	57.3% (86/150)
** Yes**	44.4% (64/150)

**Table 2:** Summary statistics for implant-level variables.

Variable	
**Deepest probing depth**	
** n**	357
** Mean ± SD**	2.42 ± 2.14
** Median**	3
** Minimum**	0
** Maximum**	10
**Mean probing depth**	
** n**	358
** Mean ± SD**	1.75 ± 1.47
** Median**	2
** Minimum**	0
** Maximum**	7.3
**Immediate implant?**	
** n**	385
** No**	44.9% (173/385)
** Yes**	55.1% (212/385)
**Restoration retention**	
** n**	88
** Screwed**	51.1% (45/88)
** Cemented**	48.9% (43/88)
**Peri-mucositis or Peri-implantitis?**	
** n**	386
** No**	74.1% (286/386)
** Yes**	25.9% (100/386)
**Purulence present**	
** n**	386
** No**	98.4% (380/386)
** Yes**	1.6% (6/386)
**Mobility**	
** n**	361
** No**	95.8% (346/361)
** Yes**	4.2% (15/361)
**Bone Graft?**	
** n**	386
** No**	54.9% (212/386)
** Yes**	45.1% (174/386)
**EMD?**	
** n**	386
** No**	80% (312/386)
** Yes**	20% (74/386)
**Bleeding on probing**	
** n**	361
** No**	91.7% (331/361)
** Yes**	8.3% (30/361)
**Implant Failure**	
** n**	386
** No**	79.0% (305/386)
** Yes**	21.0% (81/386)
**Implant Site**	
** n**	386
** Upper Molar**	18.7% (72/386)
** Upper Premolar**	21.8% (84/386)
** Upper Cuspid**	4.4% (17/386)
** Upper Incisor**	11.9% (46/386)
** Lower Molar**	23.6% (91/386)
** Lower Premolar**	12.2% (47/386)
** Lower Cuspid**	2.1% (8/386)
** Lower Incisor**	5.4% (21/386)

**Table 3:** GEE means for mean probing depth and deepest probing depth adjusted for baseline age and odds ratio for BOP adjusted for baseline age.

Variable	Mean PD ± SE	*p*	Deepest PD ± SE	*p*	OR BOP OR (95% CI)	*p*
**Baseline Age (in years)**		0.798		0.711		0.415
** 16 to <58 years**	1.70 ± 0.22		2.34 ± 0.30		1.00	
** 58 to 63 years**	1.84 ± 0.20		2.62 ± 0.30		0.96 (0.17 to 5.51)	
** 64 to 69.5 years**	1.98 ± 0.20		2.80 ± 0.32		2.94 (0.80 to 10.8)	
** ≥70 years**	1.82 ± 0.18		2.49 ± 0.25		1.68 (0.47 to 6.02)	
**Tooth Type**		0.081		0.050		0.805
** Incisor**	1.43 ± 0.17		1.90 ± 0.26		1.00	-
** Cuspid**	1.54 ± 0.22		2.09 ± 0.29		0.94 (0.15 to 6.03)	
** Premolar**	1.97 ± 0.14		2.71 ± 0.21		1.63 (0.42 to 6.32)	
** Molar**	1.91 ± 0.14		2.72 ± 0.21		1.54 (0.45 to 5.27)	
**Non-Molar or Molar**		0.417		0.202		0.750
** Non-Molar**	1.78 ± 0.12		2.43 ± 0.17		1.00	
** Molar**	1.88 ± 0.13		2.67 ± 0.21		1.12 (0.53 to 2.41)	
**Arch**		0.211		0.254		0.766
** Mandibular**	1.72 ± 0.13		2.40 ± 0.20		1.00	
** Maxillary**	1.91 ± 0.13		2.66 ± 0.19		0.88 (0.37 to 2.11)	
**Smoking Status**		0.364		0.236		0.660
** Non-Smoker**	1.76 ± 0.13		2.45 ± 0.20		1.00	
** Smoker**	2.03 ± 0.26		2.96 ± 0.38		0.79 (0.27 to 2.35)	
**Diabetes Mellitus**		0.041		0.058		0.497
** No**	1.99 ± 0.23		2.46 ± 0.17		1.00	
** Yes**	2.58 ± 0.06		3.88 ± 0.61		2.20 (0.48 to 10.2)	
**Hygiene Status**		0.196		0.140		0.947
** Satisfactory**	1.78 ± 0.11		2.46 ± 0.17		1.00	
** Unsatisfactory**	2.71 ± 0.66		4.26 ± 1.09		0.93 (0.13 to 6.86)	
**Periodontal Disease**		0.004		0.003		0.287
** No**	1.34 ± 0.14		1.85 ± 0.20		1.00	
** Yes**	2.58 ± 0.36		3.84 ± 0.57		2.17 (0.67 to 7.06)	
**Immediate Implant**		0.124		0.256		0.745
** No**	1.69 ± 0.14		2.41 ± 0.21		1.00	
** Yes**	1.97 ± 0.15		2.70 ± 0.22		0.87 (0.37 to 2.03)	
**Cemented or Screwed**		0.327		0.228		0.662
** Cemented**	2.04 ± 0.24		3.03 ± 0.46		1.00	
** Screwed**	1.79 ± 0.24		2.42 ± 0.43		1.38 (0.32 to 5.90)	
**Nightguard**		0.142		0.369		0.077
** No**	2.01 ± 0.15		2.41 ± 0.25		1.00	
** Yes**	1.67 ± 0.16		2.70 ± 0.21		0.44 (0.17 to 1.10)	
**Peri-implantitis/peri-mucositis**		<0.001		<0.001		0.003
** No**	1.64 ± 0.10		2.24 ± 0.15		1.00	
** Yes**	2.51 ± 0.28		3.75 ± 0.36		14.0 (2.61 to 25.4)	
**Presence of Purulence**		0.025		0.028		0.107
** No**	1.76 ± 0.11		2.44 ± 0.16		1.00	
** Yes**	4.39 ± 0.50		6.89 ± 0.95		14.0 (2.61 to 75.4)	
**Bone Graft**		0.893		0.595		0.284
** No**	1.84 ± 0.15		2.62 ± 0.22		1.00	
** Yes**	1.81 ± 0.15		2.47 ± 0.21		0.63 (0.26 to 1.54)	
**EMD**		0.721		0.933		0.498
** No EMD**	1.79 ± 0.15		2.55 ± 0.22		1.00	
** EMD**	1.85 ± 0.14		2.53 ± 0.20		0.73 (0.29 to 1.82)	
**EMD + Bone Graft?**		0.867		0.850		0.611
** No EMD + No Bone Graft**	1.81 ± 0.16		2.59 ± 0.23		1.00	
** No EMD + Bone Graft**	1.76 ± 0.30		2.44 ± 0.41		0.60 (0.19 to 1.87)	
** EMD + No Bone Graft**	2.00 ± 0.23		2.77 ± 0.30		0.92 (0.18 to 4.74)	
** EMD + Bone Graft**	1.82 ± 0.15		2.48 ± 0.23		0.63 (0.22 to 1.73)	

**Table 4:** Relative risk for individual factors associated with implant failure with all models adjusted for baseline age in categories. Frailty survival models were fit for all models.

Variable	Relative Risk	95% CI	*p*
**Age**			0.040
** 16 to <58 Years**	reference	-	
** 58 to <68 Years**	2.32	1.20 to 4.49	
** 68 to 88 Years**	1.94	0.82 to 4.63	
**Gender**			0.589
** Female**	reference	-	
** Male**	1.21	0.61 to 2.39	
**Smoking Status**			0.069
** Nonsmoker**	reference	-	
** Smoker**	2.12	0.95 to 4.73	
**Diabetes Mellitus**			0.019
** No**	reference	-	
** Yes**	3.10	1.01 to 9.46	
**Immediate Implant**			0.674
** No**	reference	-	
** Yes**	1.13	0.65 to 1.97	
**EMD**			0.241
** No EMD**	reference	-	
** EMD**	1.36	0.76 to 2.46	
**DFDBA**			0.190
** No**	reference	-	
** Yes**	0.71	0.40 to 1.25	
**Deepest Probing Depth**			0.845
** 3 mm or less**	reference	-	
** >3 mm**	0.85	0.41 to 1.73	
**Mean Probing Depth**			0.185
** 2 mm or less**	reference	-	
** >2 mm**	0.63	0.32 to 1.24	
**Nightguard**			0.282
** No**	reference	-	
** Yes**	0.70	0.36 to 1.35	
**Oral Hygiene**			0.031
** Satisfactory**	reference	-	
** Unsatisfactory**	3.15	1.11 to 8.90	
**Periodontitis?**			0.014
** No Periodontitis**	reference	-	
** Periodontitis**	2.45	1.06 to 5.64	
**Presence of Purulence?**			0.231
** No Purulence**	reference	-	
** Purulence**	2.22	0.60 to 8.22	
**Presence of Peri-mucositis/Peri-implantitis?**			<0.001
** No Peri-mucositis/Peri-implantitis**	reference	-	
** Peri-mucositis/Peri-implantitis**	5.94	3.12 to 11.3	
**Tooth Type**			0.913
** Incisor**	reference	-	
** Cuspid**	0.78	0.21 to 2.90	
** Premolar**	0.88	0.40 to 1.92	
** Molar**	1.02	0.48 to 2.20	
**Molar Status**			0.659
** Non-Molar**	reference	-	
** Molar**	1.13	0.66 to 1.94	
**Arch**			0.144
** Mandibular**	reference	-	
** Maxillary**	0.64	0.36 to 1.16	
**EMD and Bone Graft**			0.115
** No EMD and No Bone Graft**	reference	-	
** No EMD and Bone Graft**	0.31	0.10 to 0.95	
** EMD and No Bone Graft**	1.74	0.68 to 4.45	
** EMD and Bone Graft**	1.06	0.55 to 2.04	

**Table 5:** Relative risk for individual factors associated with implant failure with all models adjusted for baseline age as a continuous variable. Frailty survival models were fit for all models.

Variable	Relative Risk	95% CI	*p*
**Age**			0.117
** (continuous—per year)**	1.02	1.00 to 1.05	
**Gender**			0.474
** Female**	reference	-	
** Male**	1.28	0.66 to 2.48	
**Smoking Status**			0.075
** Nonsmoker**	reference	-	
** Smoker**	3.18	0.93 to 4.47	
**Diabetes Mellitus**			0.060
** No**	reference	-	
** Yes**	2.86	0.96 to 8.56	
**Immediate Implant**			0.715
** No**	reference	-	
** Yes**	1.11	0.64 to 1.94	
**EMD**			0.297
** No EMD**	reference	-	
** EMD**	1.37	0.76 to 2.47	
**Bone Graft**			0.234
** No**	reference	-	
** Yes**	0.71	0.40 to 1.25	
**Deepest Probing Depth**			0.539
** 3 mm or less**	reference	-	
** >3 mm**	0.80	0.39 to 1.63	
**Mean Probing Depth**			0.210
** 2 mm or less**	reference	-	
** >2 mm**	0.66	0.34 to 1.27	
**Nightguard**			0.354
** No**	reference	-	
** Yes**	0.74	0.38 to 1.41	
**Oral Hygiene**			0.011
** Satisfactory**	reference	-	
** Unsatisfactory**	3.01	1.10 to 8.23	
**Periodontitis?**			0.063
** No Periodontitis**	reference	-	
** Periodontitis**	2.19	0.96 to 5.00	
**Presence of Purulence?**			0.134
** No Purulence**	reference	-	
** Purulence**	2.67	0.74 to 9.64	
**Presence of Peri-mucositis/Peri-implantitis?**			<0.001
** No Peri-mucositis/Peri-implantitis**	reference	-	
** Peri-mucositis/Peri-implantitis**	5.55	2.95 to 10.4	
**Tooth Type**			0.961
** Incisor**	reference	-	
** Cuspid**	0.79	0.21 to 2.94	
** Premolar**	0.84	0.38 to 1.84	
** Molar**	0.92	0.43 to 1.96	
**Molar Status**			0.865
** Non-Molar**	reference	-	
** Molar**	1.05	0.62 to 1.78	
**Arch**			0.307
** Mandibular**	reference	-	
** Maxillary**	0.75	0.42 to 1.31	
**EMD and Bone Graft**			0.107
** No EMD and No Bone Graft**	reference	-	
** No EMD and Bone Graft**	0.31	0.10 to 0.95	
** EMD and No Bone Graft**	1.78	0.71 to 4.50	
** EMD and Bone Graft**	1.06	0.56 to 2.04	

**Table 6:** Survival models for age categories by EMD category, deepest probing depth category, and mean probing depth category.

	No EMD				EMD		
**Variable**	**Relative Risk**	**95% CI**	***p***	**Variable**	**Relative Risk**	**95% CI**	***p***
**Age**			0.047	**Age**			0.557
** 16 to 58 Years**	reference	-		** 16 to 58 Years**	reference	-	
** >58 to 68 Years**	2.75	1.20 to 6.29		** >58 to 68 Years**	1.24	0.41 to 3.74	
** >68 to 88 Years**	3.14	0.34 to 3.80		** >68 to 88 Years**	1.98	0.56 to 7.01	
	**Deepest Probing Depth ≤ ** **3 mm**				**Deepest Probing Depth > 3 mm**		
**Variable**	**Relative Risk**	**95% CI**	***p***	**Variable**	**Relative Risk**	**95% CI**	***p***
**Age**			0.286	**Age**			0.120
** 16 to 58 Years**	reference	-		** 16 to 58 Years**	reference	-	
** >58 to 68 Years**	1.94	0.85 to 4.44		** >58 to 68 Years**	6.90	1.08 to 44.1	
** >68 to 88 Years**	1.61	0.51 to 5.04		** >68 to 88 Years**	4.02	0.53 to 30.6	
	**Mean Probing Depth ≤ 2** ** mm**				**Mean Probing Depth > 2 mm**		
**Variable**	**Relative Risk**	**95% CI**	***p***	**Variable**	**Relative Risk**	**95% CI**	***p***
**Age**			0.116	**Age**			0.400
** 16 to 58 Years**	reference	-		** 16 to 58 Years**	reference	-	
** >58 to 68 Years**	2.60	1.02 to 6.62		** >58 to 68 Years**	2.44	0.63 to 2.43	
** >68 to 88 Years**	2.39	0.65 to 8.72		** >68 to 88 Years**	2.23	0.47 to 10.7	

## Data Availability

The original contributions presented in this study are included in the article. Further inquiries can be directed to the corresponding author.

## References

[1] Becker W., Hujoel P., Becker B.E., Wohrle P. (2016). Dental Implants in an Aged Population: Evaluation of Periodontal Health, Bone Loss, Implant Survival, and Quality of Life. Clin. Implant. Dent. Relat. Res..

[2] Becker W., Hujoel P.P., Becker B.E., Willingham H. (2000). Osteoporosis and implant failure: An exploratory case-control study. J. Periodontol..

[3] Buser D., Janner S.F., Wittneben J.G., Brägger U., Ramseier C.A., Salvi G.E. (2012). 10-year survival and success rates of 511 titanium implants with a sandblasted and acid-etched surface: A retrospective study in 303 partially edentulous patients. Clin. Implant. Dent. Relat. Res..

[4] Van Velzen F.J., Ofec R., Schulten E.A., Ten Bruggenkate C.M. (2015). 10-year survival rate and the incidence of peri-implant disease of 374 titanium dental implants with a SLA surface: A prospective cohort study in 177 fully and partially edentulous patients. Clin. Oral Implant. Res..

[5] Shimchuk A.A., Weinstein B.F., Daubert D.M. (2021). The impact of a change in classification criteria on the prevalence of peri-implantitis: A cross-sectional analysis. J. Periodontol..

[6] Diaz P., Gonzalo E., Villagra L.J.G., Miegimolle B., Suarez M.J. (2022). What is the prevalence of peri-implantitis? A systematic review and meta-analysis. BMC Oral Health.

[7] Derks J., Schaller D., Håkansson J., Wennström J.L., Tomasi C., Berglundh T. (2016). Effectiveness of Implant Therapy Analyzed in a Swedish Population: Prevalence of Peri-implantitis. J. Dent. Res..

[8] Heitz-Mayfield L.J., Mombelli A. (2014). The therapy of peri-implantitis: A systematic review. Int. J. Oral Maxillofac. Implant..

[9] Khoury F., Keeve P.L., Ramanauskaite A., Schwarz F., Koo K.T., Sculean A., Romanos G. (2019). Surgical treatment of peri-implantitis—Consensus report of working group 4. Int. Dent. J..

[10] Roccuzzo M., Mirra D., Roccuzzo A. (2024). Surgical treatment of peri-implantitis. Br. Dent. J..

[11] Schwarz F., Alcoforado G., Guerrero A., Jönsson D., Klinge B., Lang N., Mattheos N., Mertens B., Pitta J., Ramanauskaite A. (2021). Peri-implantitis: Summary and consensus statements of group 3. The 6th EAO Consensus Conference 2021. Clin. Oral Implant. Res..

[12] Di Gianfilippo R., Sirinirund B., Rodriguez M.V., Chen Z., Wang H.-L. (2020). Long-Term Prognosis of Peri-Implantitis Treatment: A Systematic Review of Prospective Trials with More Than 3 Years of Follow-Up. Appl. Sci..

[13] Kourtis S.G., Sotiriadou S., Voliotis S., Challas A. (2004). Private practice results of dental implants. Part I: Survival and evaluation of risk factors—Part II: Surgical and prosthetic complications. Implant. Dent..

[14] Solderer A., Paterno Holtzman L., Milinkovic L., Pitta J., Malpassi C., Wiedemeier D., Cordaro L. (2024). Implant failure and clinical and radiographic outcomes after surgical treatment of peri-implantitis: A meta-analysis. Int. J. Oral Implantol..

[15] Wilson T.G., Harrel S.K., Nunn M.E. (2023). The Use of Enamel Matrix Derivative during Surgical Therapy for Peri-Implantitis: A Case Series. Dent. J..

[16] Nyland A.N., Lie S.A., Gjerde C.G. (2024). Risk Factors for Early Dental Implant Failure. Int. J. Oral Maxillofac. Implant..

[17] Do T.A., Le H.S., Shen Y.W., Huang H.L., Fuh L.J. (2020). Risk Factors related to Late Failure of Dental Implant-A Systematic Review of Recent Studies. Int. J. Environ. Res. Public Health.

[18] Chen H., Liu N., Xu X., Qu X., Lu E. (2013). Smoking, radiotherapy, diabetes and osteoporosis as risk factors for dental implant failure: A meta-analysis. PLoS ONE.

[19] Yari A., Fasih P., Alborzi S., Nikzad H., Romoozi E. (2024). Risk factors associated with early implant failure: A retrospective review. J. Stomatol. Oral Maxillofac. Surg..

[20] Schimmel M., Srinivasan M., McKenna G., Müller F. (2018). Effect of advanced age and/or systemic medical conditions on dental implant survival: A systematic review and meta-analysis. Clin. Oral Implant. Res..

[21] Boboeva O., Kwon T.G., Kim J.W., Lee S.T., Choi S.Y. (2021). Comparing factors affecting dental-implant loss between age groups: A retrospective cohort study. Clin. Implant. Dent. Relat. Res..

[22] Zhao H., Fan S., Sun J. (2024). Delayed wound healing in the elderly and a new therapeutic target: CD271. Curr. Stem Cell Res. Ther..

[23] Elloso M., Kambli A., Aijaz A., van de Kamp A., Jeschke M.G. (2020). Burns in the Elderly: Potential Role of Stem Cells. Int. J. Mol. Sci..

[24] D’Ippolito G., Schiller P.C., Ricordi C., Roos B.A., Howard G.A. (1999). Age-related osteogenic potential of mesenchymal stromal stem cells from human vertebral bone marrow. J. Bone Miner. Res..

[25] Kanasi E., Ayilavarapu S., Jones J. (2016). The aging population: Demographics and the biology of aging. Periodontol. 2000.

[26] Liu J., Zhang J., Lin X., Boyce B.F., Zhang H., Xing L. (2022). Age-associated callus senescent cells produce TGF-β1 that inhibits fracture healing in aged mice. J. Clin. Investig..

[27] Mi B., Xiong Y., Knoedler S., Alfertshofer M., Panayi A.C., Wang H., Lin S., Li G., Liu G. (2024). Ageing-related bone and immunity changes: Insights into the complex interplay between the skeleton and the immune system. Bone Res..

[28] Renvert S., Berglund J., Persson R.E., Persson G.R. (2011). Osteoporosis and periodontitis in older subjects participating in the Swedish National Survey on Aging and Care (SNAC-Blekinge). Acta Odontol. Scand..

[29] Müller F., Srinivasan M., Krause K.H., Schimmel M. (2022). Periodontitis and peri-implantitis in elderly people experiencing institutional and hospital confinement. Periodontol. 2000.

[30] Vaughan R. (2017). Oversampling in Health Surveys: Why, When, and How?. Am. J. Public Health.

[31] Fawzy El-Sayed K.M., Dörfer C., Ungefroren H., Kassem N., Wiltfang J., Paris S. (2014). Effect of Emdogain enamel matrix derivative and BMP-2 on the gene expression and mineralized nodule formation of alveolar bone proper-derived stem/progenitor cells. J. Craniomaxillofac. Surg..

[32] Li G., Hu J., Chen H., Chen L., Zhang N., Zhao L., Wen N., Yang Y. (2017). Enamel matrix derivative enhances the proliferation and osteogenic differentiation of human periodontal ligament stem cells on the titanium implant surface. Organogenesis.

[33] Li Y., Cheng L., Xia Q., Meng M., Wang Q., Zeng X., Jia Y., Liu C., Wu L., Chen H. (2023). Synergistic effects of enamel matrix derivatives and surface morphology of anodized titanium on osteogenic differentiation of bone marrow mesenchymal stem cells sheet. Am. J. Transl. Res..

[34] Schwarz F., Derks J., Monje A., Wang H.L. (2018). Peri-implantitis. J. Periodontol..

[35] Rethman M.P., Nunn M.E. (1999). Clinical versus statistical significance. J. Periodontol..

[36] Greenstein G., Nunn M.E. (2004). A method to enhance determining the clinical relevance of periodontal research data: Number needed to treat (NNT). J. Periodontol..

[37] Yang Y., Khorshidi H.A., Aickelin U. (2024). A review on over-sampling techniques in classification of multi-class imbalanced datasets: Insights for medical problems. Front. Digit. Health.

